# Hedgehog signaling in the airway epithelium of patients with chronic obstructive pulmonary disease

**DOI:** 10.1038/s41598-019-40045-3

**Published:** 2019-03-04

**Authors:** A. Tam, M. Hughes, K. M. McNagny, M. Obeidat, T. L. Hackett, J. M. Leung, T. Shaipanich, D. R. Dorscheid, G. K. Singhera, C. W. T. Yang, P. D. Paré, J. C. Hogg, D. Nickle, D. D. Sin

**Affiliations:** 1 Center for Heart Lung Innovation, St. Paul’s Hospital, Vancouver, British Columbia Canada; 2 Biomedical Research Centre (BRC), University of British Columbia, Vancouver, British Columbia Canada; 3 Department of Anaesthesiology, Pharmacology, & Therapeutics, University of British Columbia, Vancouver, British Columbia Canada; 4 Division of Respiratory Medicine, Department of Medicine, St. Paul’s Hospital, University of British Columbia, Vancouver, British Columbia Canada; 5 Merck & Co. Inc., Rahway, New Jersey United States of America

## Abstract

Genome-wide association studies have linked gene variants of the receptor patched homolog 1 (*PTCH1*) with chronic obstructive pulmonary disease (COPD). However, its biological role in the disease is unclear. Our objective was to determine the expression pattern and biological role of *PTCH1* in the lungs of patients with COPD. Airway epithelial-specific PTCH1 protein expression and epithelial morphology were assessed in lung tissues of control and COPD patients. *PTCH1* mRNA expression was measured in bronchial epithelial cells obtained from individuals with and without COPD. The effects of *PTCH1* siRNA knockdown on epithelial repair and mucous expression were evaluated using human epithelial cell lines. *Ptch1*^+/−^ mice were used to assess the effect of decreased PTCH1 on mucous expression and airway epithelial phenotypes. Airway epithelial-specific PTCH1 protein expression was significantly increased in subjects with COPD compared to controls, and its expression was associated with total airway epithelial cell count and thickness. *PTCH1* knockdown attenuated wound closure and mucous expression in airway epithelial cell lines. *Ptch1*^+/−^ mice had reduced mucous expression compared to wildtype mice following mucous induction. PTCH1 protein is up-regulated in COPD airway epithelium and may upregulate mucous expression. PTCH1 provides a novel target to reduce chronic bronchitis in COPD patients.

## Introduction

Chronic obstructive pulmonary disease (COPD) is a highly prevalent, progressive disorder that affects over 300 million people and accounts for 3 million deaths per year worldwide^1^. Its pathophysiology, however, is not well known and there are no therapies that can modify its disease progression. Recently, large-scale genome-wide association studies (GWAS) have been used to identify potential molecular drivers of COPD^2–8^. These GWAS data along with follow up integrative genomics studies in lung tissues have implicated members of the hedgehog signalling pathway including the hedgehog interacting protein (HHIP) and the receptor for the pathway patched homolog 1 (PTCH1) as important drivers of COPD and lung function variation^2^. Activation of the hedgehog signalling pathway is involved in embryonic lung development and plays an active role in adult diseases such as pulmonary fibrosis, asthma, COPD and lung cancer^9,10^. PTCH1 is a transmembrane protein receptor for the secreted hedgehog ligands including sonic hedgehog (SHH), indian hedgehog (IHH) and desert hedgehog (DHH). In the absence of hedgehog ligand binding, PTCH1 inhibits signalling through the G-protein-coupled receptor smoothened (SMO). In the presence of hedgehog ligand binding, however, PTCH1 dissociates from SMO, which results in downstream activation of a family of zinc-finger DNA-binding proteins (GLI)-related transcription factors^9^. Activation of hedgehog signalling has been shown to induce transcription of proliferative genes, as well as *PTCH1* which act in a negative feedback loop to turn off hedgehog signalling^9,11,12^. In contrast, inhibition of hedgehog signaling has been shown to be implicated in decreased proliferation in human mesenchymal stem cells that is not linked to apoptosis but by arresting cells in the G0/G1 phases of the cell cycle^13^. Intriguingly, activation of hedgehog signaling in neuronal progenitors cells affected cell division, cell cycle length and cell growth, which may have important implications on cell proliferation and differentiation^14^.

The exact expression pattern of PTCH1 protein in the lungs of patients with COPD and its biological role has not been thoroughly investigated. We hypothesize that hedgehog signalling is dysregulated in COPD patients which, in turn, may lead to increased cell proliferation and mucous expression in the airways. These endpoints are of great interest in COPD as currently there are no therapies that reduce mucous production and cough, which are symptoms of great importance to patients^15^.

In this study, we assessed the protein expression levels and cell types in which PTCH1 is expressed in the airway epithelium of patients with and without COPD, and the biological role of PTCH1 on cell proliferation and mucous expression *in vitro* and *in vivo*.

## Results

### PTCH1 protein is up-regulated in the airway epithelium of patients with COPD compared to subjects without COPD

**Figure 1:**
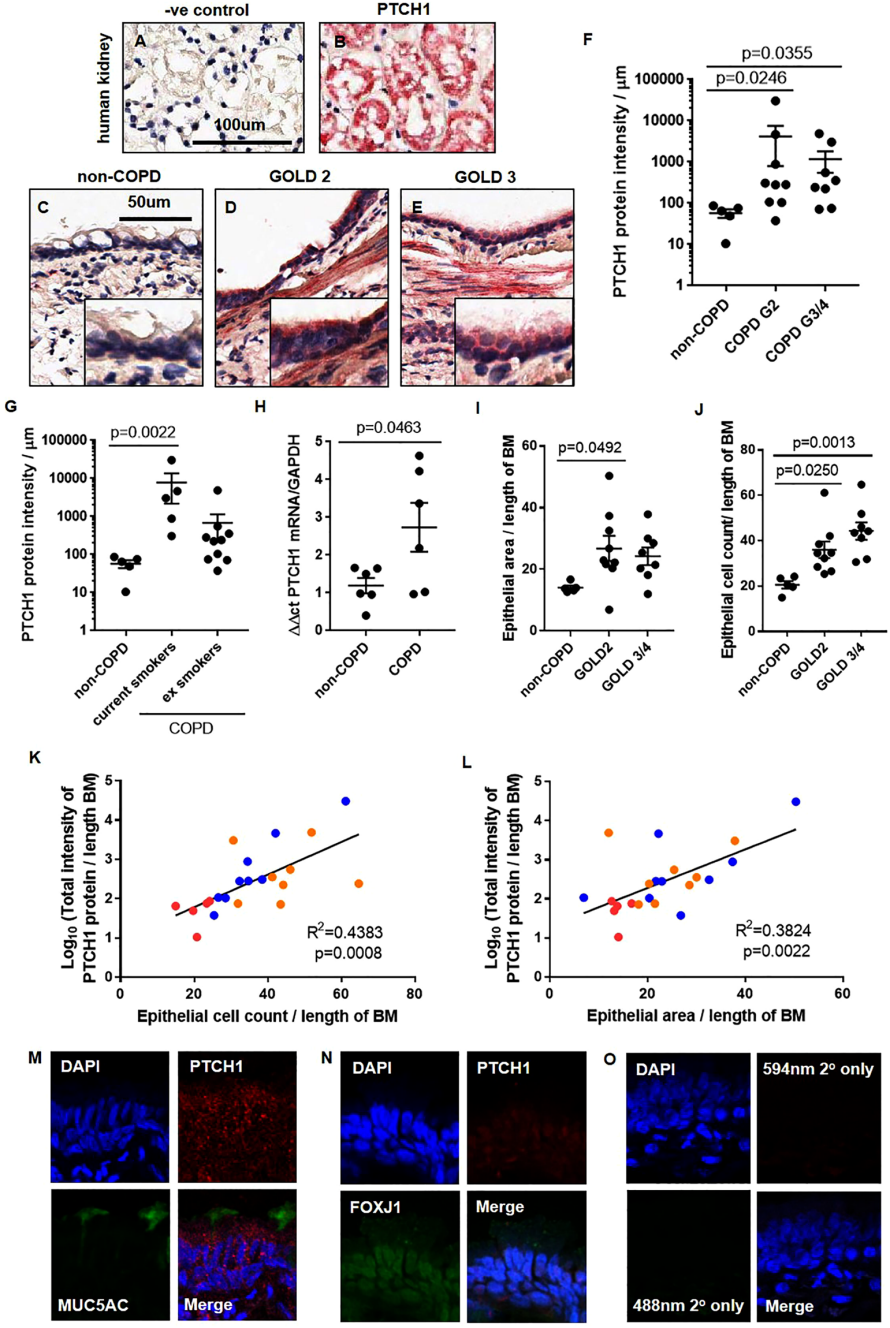
PTCH1 protein is up-regulated in the airway epithelium of patients with COPD compared to subjects without COPD. Paraffin-embedded human kidney tissues stained with (**A**) secondary only and (**B**) PTCH1 antibody were shown. Paraffin-embedded human lung tissues from subjects (**C**) without COPD, (**D**) COPD GOLD STAGE 2, and (**E**) COPD GOLD STAGE 3 were stained with PTCH1 antibody. Airway epithelium-specific PTCH1 protein expression was normalized to the length of basement membrane (µm) in (**F**) non-COPD, COPD GOLD STAGE 2 and GOLD STAGE 3/4, and (**G**) with COPD stratified by smoking status (current vs ex-smokers). (**H**) *PTCH1* mRNA expression was normalized to GAPDH and expressed as ∆∆ct in human bronchial brushings from subjects with or without COPD. (**I**) Airway epithelial thickness and (**J**) total airway epithelial cell counts were quantified in subjects without COPD, COPD GOLD STAGE 2 and GOLD STAGE 3/4. Values were expressed as mean ± SEM. Kruskal–Wallis test with Dunnett’s multiple comparisons test was used in panels F,G. A two-tailed unpaired parametric t test was used in panel H. One-way analysis of variance was used in panels I,J. Correlations between total epithelial-specific PTCH1 protein expression (data log-transformed) with (**K**) epithelial thickness and (**L**) total epithelial cell count were shown. Linear regression analyses were used in panels K and L. Red dot = non-COPD, blue dot = COPD GOLD STAGE 2, orange dot = COLD GOLD STAGE 3/4. Representative immunofluorescence images of lung tissues from a patient with COPD GOLD STAGE 2 stained with (**M**) PTCH1 and MUC5AC (goblet cell) antibodies, (**N**) PTCH1 and FOXJ1 (ciliated cell) and (**O**) secondary only were shown.

To determine the expression pattern of hedgehog signalling receptor (PTCH1) in lung tissues of patients with COPD, formalin-fixed paraffin-embedded (FFPE) lung tissues from COPD patients and healthy controls were evaluated by immunohistochemistry (IHC). FFPE-human kidney tissues were used as positive control for PTCH1 protein staining by IHC (Fig. 1A,B). Subject demographics and spirometric measurements are shown in Tables 1,2. As shown in Fig. 1, we saw a striking increase in PTCH1 protein in the airway epithelium of subjects with COPD GOLD STAGE 2 and GOLD STAGE 4 when compared to controls (see Fig. 1C–E for representative examples and Fig. 1F for quantification). Intriguingly, current smokers with COPD had significantly higher PTCH1 protein expression in the airway epithelium than ex-smokers with COPD (Fig. 1G). Consistently, *PTCH1* mRNA expression was significantly increased in epithelial cells obtained by bronchial-brushings from patients with COPD compared to subjects without COPD (Fig. 1H). To test whether PTCH1 expression is associated with epithelial status in COPD patient tissues, we assessed airway epithelial thickness and total airway epithelial cell count with respect to PTCH1 expression (Fig. 1I,J). As expected, in COPD patients, each of these parameters was increased compared to healthy controls. Similarly, we observed a significant positive correlation between the level of PTCH1 expression and epithelial area as well as total epithelial cell count (normalized to basement membrane length) (Fig. 1K,L). Using immunofluorescence microscopy, we showed that PTCH1 protein was co-expressed with MUC5AC (mucous-producing cells) and FOXJ1 (ciliated cells) in a representative COPD GOLD STAGE 2 FFPE-lung tissue. (Fig. 1M,N). Tissue-matched negative control section stained with secondary antibodies was shown in Fig. 1O.

**Table 1:** Demographic characteristics of COPD patients and control subjects donating tissues for immunohistochemical analysis.

Characteristic	non-COPD	GOLD STAGE 2	GOLD STAGE 3,4
Sex (M/F)	2/3	6/3	3/5
Smoking status (non/current/ex/NA)	5/0/0/0	0/4/3/2	0/1/7/0
Age (mean ± SD)	59.6 ± 19.8	63.7 ± 9.0	61.0 ± 6.1
%FEV1/FVC (mean ± SD)	83.0 ± 4.4	57.1 ± 5.6	33.4 ± 11.9

**Table 2:** Demographic characteristics of study patients and controls donating tissues for gene expression analysis.

Characteristic	non-COPD	COPD
Sex (M/F)	2/4	4/2
Smoking status (non/current/ex)	3/1/2	0/4/2
Age (mean ± SD)	59.3 ± 14.5	66.8 ± 8.8
%FEV1/FVC (mean ± SD)	81.6 ± 10.4	60.1 ± 12.6

### Total epithelial cell count positively correlates with airway mucous expression and cell proliferation

**Figure 2:**
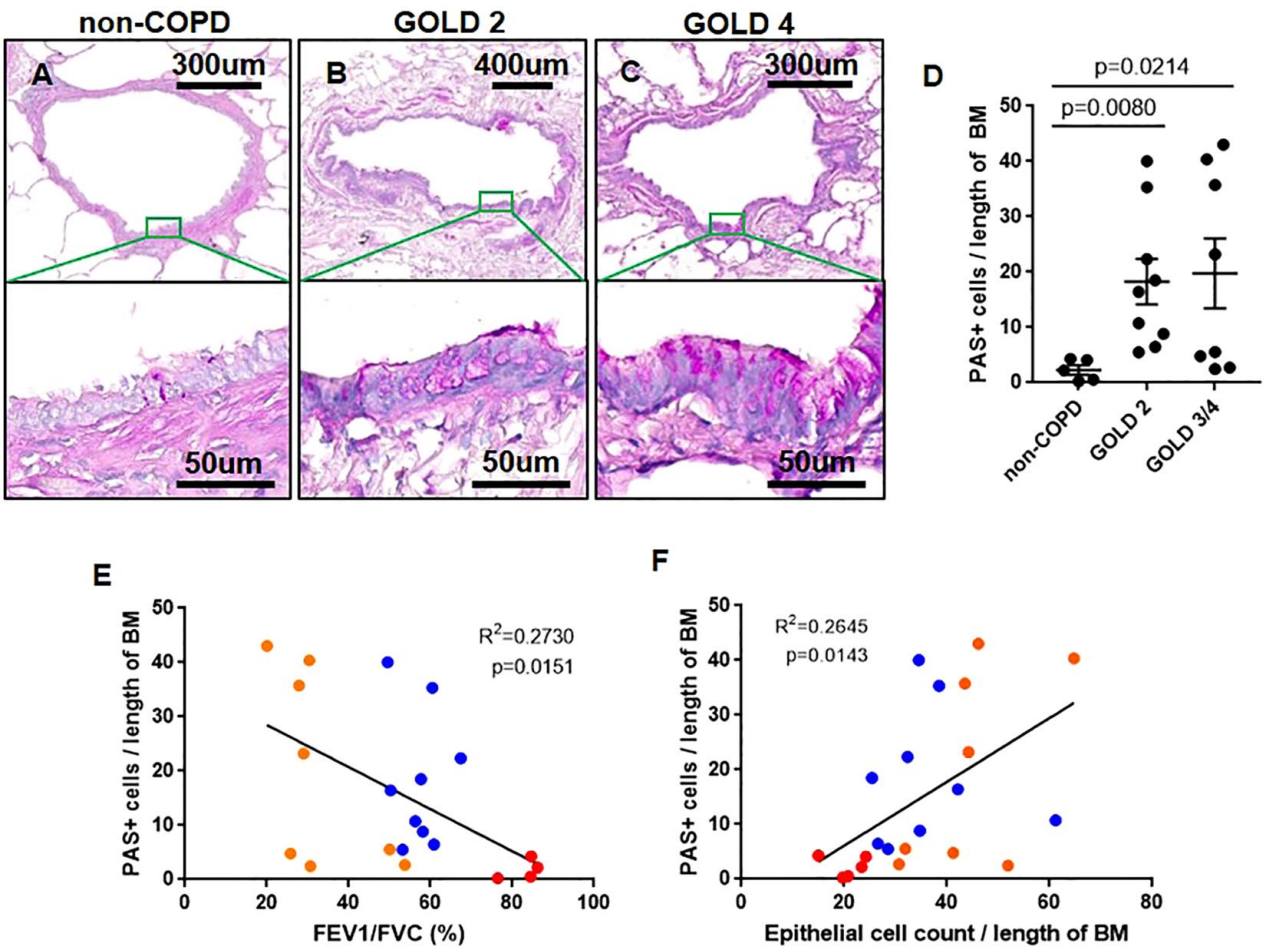
COPD airways have increased mucous expression and epithelial cell count. Representative image of human lung tissues from (**A**) non-COPD, (**B**) COPD GOLD STAGE 2, and (**C**) COPD GOLD STAGE 4 stained with periodic acidic Schiff (PAS) for total carbohydrate content on mucous expression in the airway epithelium were shown. (**D**) Airway epithelial-specific PAS+ cells were normalized to the length of basement membrane in subjects without COPD, COPD GOLD STAGE 2 and GOLD STAGE 3/4. Correlations between airway epithelial-specific PAS+ cells with (**E**) FEV1/FVC (%) and (**F**) total epithelial cell count were shown. The Kruskal–Wallis test with Dunnett’s multiple comparisons test was used in panel D. Linear regression analyses were used in panels E and F. Red dot = non-COPD, blue dot = COPD GOLD STAGE 2, orange dot = COLD GOLD STAGE 3/4. (Note: one non-COPD subject has missing FEV1/FVC value).

To determine whether mucous expression is increased with COPD severity, FFPE-lung tissues were stained with periodic acid Schiff (PAS) for total glycoproteins and evaluated for mucous-producing cells in the airway epithelium (Fig. 2A–C). PAS positive (+) cells were significantly increased in patients with COPD compared to non-COPD subjects (Fig. 2D). PAS+ cells were negatively associated with FEV_1_/FVC (Fig. 2E). Interestingly, we observed a significant positive correlation between the number of PAS+ cells and total epithelial cell count after normalization to basement membrane length (Fig. 2F).

**Figure 3:**
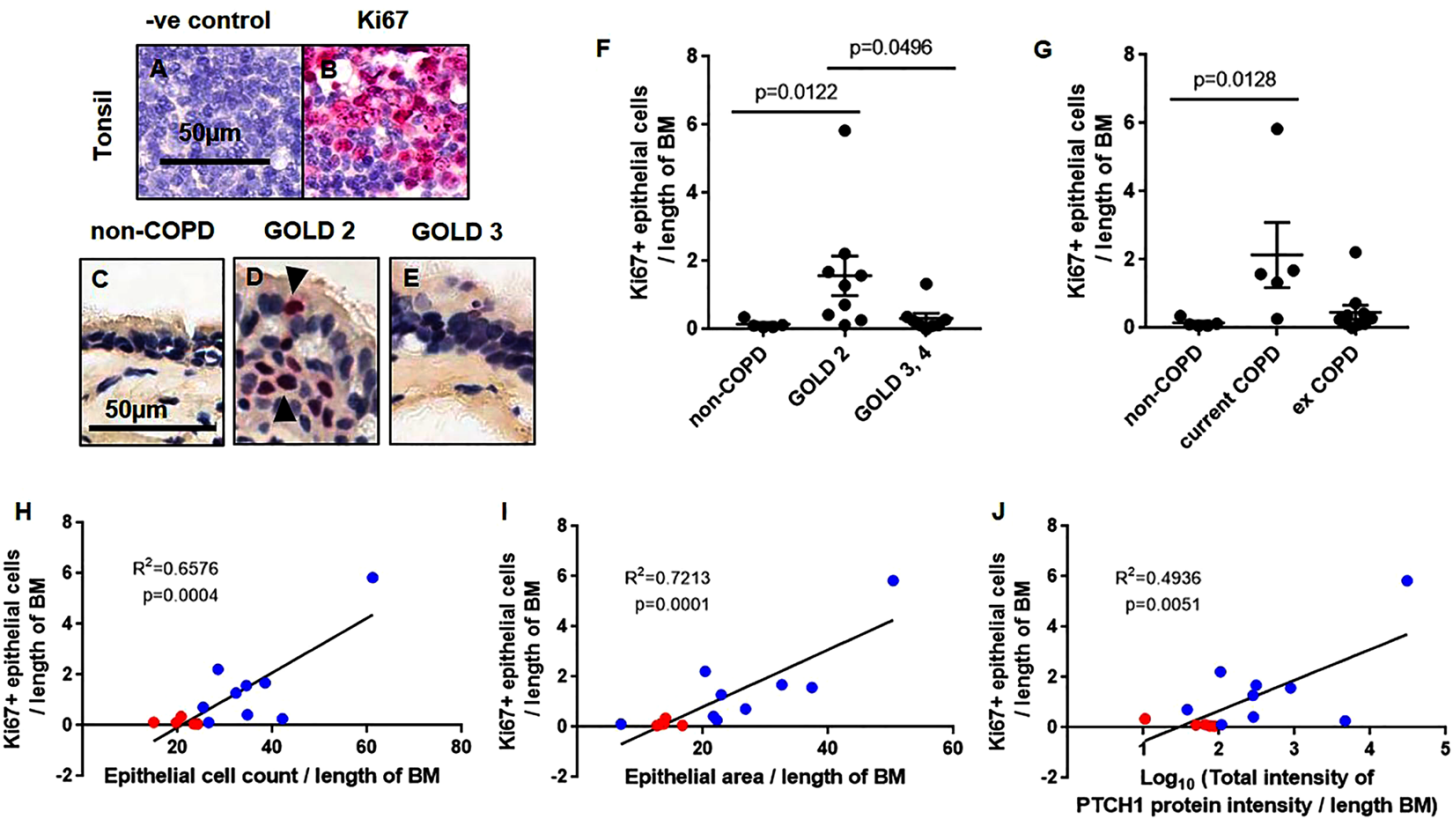
Index of epithelial cell proliferation positively correlate with PTCH1 protein in moderate COPD patients. (**A**,**B**) FFPE-human tonsil sections were stained with Ki67 as an index of cellular proliferation. Representative image of human lung tissues from (**C**) non-COPD, (**D**) COPD GOLD STAGE 2, and (**E**) COLD GOLD STAGE 3 stained with Ki67 antibody were shown. Airway epithelial-specific Ki67+ cells was normalized to the length of basement membrane (µm) in (**F**) non-COPD, COPD GOLD STAGE 2 and GOLD STAGE 3,4, and (**G**) with COPD stratified by smoking status (current vs ex-smokers). Values were expressed as mean ± SEM in panels F and G. The Kruskal–Wallis test with Dunnett’s multiple comparisons test was used in panel F and G. Correlations between airway epithelial-specific Ki67+ cells with (**H**) total epithelial cell count, (**I**) epithelial thickness and (**J**) total epithelial-specific PTCH1 protein expression (data log-transformed) were shown. Linear regression analyses were used in panels H–J. Red dot = non-COPD, blue dot = COPD GOLD STAGE 2.

To determine an index of cellular proliferation in the airway epithelium, FFPE-lung sections were stained for expression of the proliferation associated antigen, Ki67. Highly proliferative human tonsil tissues were used as positive control (Fig. 3A,B). Representative images of lung section stained for Ki67 protein in the airway epithelium of non-COPD, COPD GOLD STAGE 2 and GOLD STAGE 3 are shown in Fig. 3C–E. Interestingly, Ki67+ cell count was significantly increased in the airway epithelium of patients with COPD GOLD STAGE 2 but not in GOLD STAGE 3,4 compared to non-COPD subjects (Fig. 3F). After stratification by smoking status, Ki67+ cell count was consistently elevated in current smokers but not ex-smokers with COPD compared to non-COPD subjects (Fig. 3G).

In subjects without COPD and COPD GOLD STAGE 2, Ki67+ cell count in the airway epithelium was positively correlated with total epithelial cell count, epithelial thickness and airway epithelial-specific PTCH1 protein expression, respectively (Fig. 3H–J).

### *PTCH1* gene silencing reduces cell proliferation and attachment

**Figure 4:**
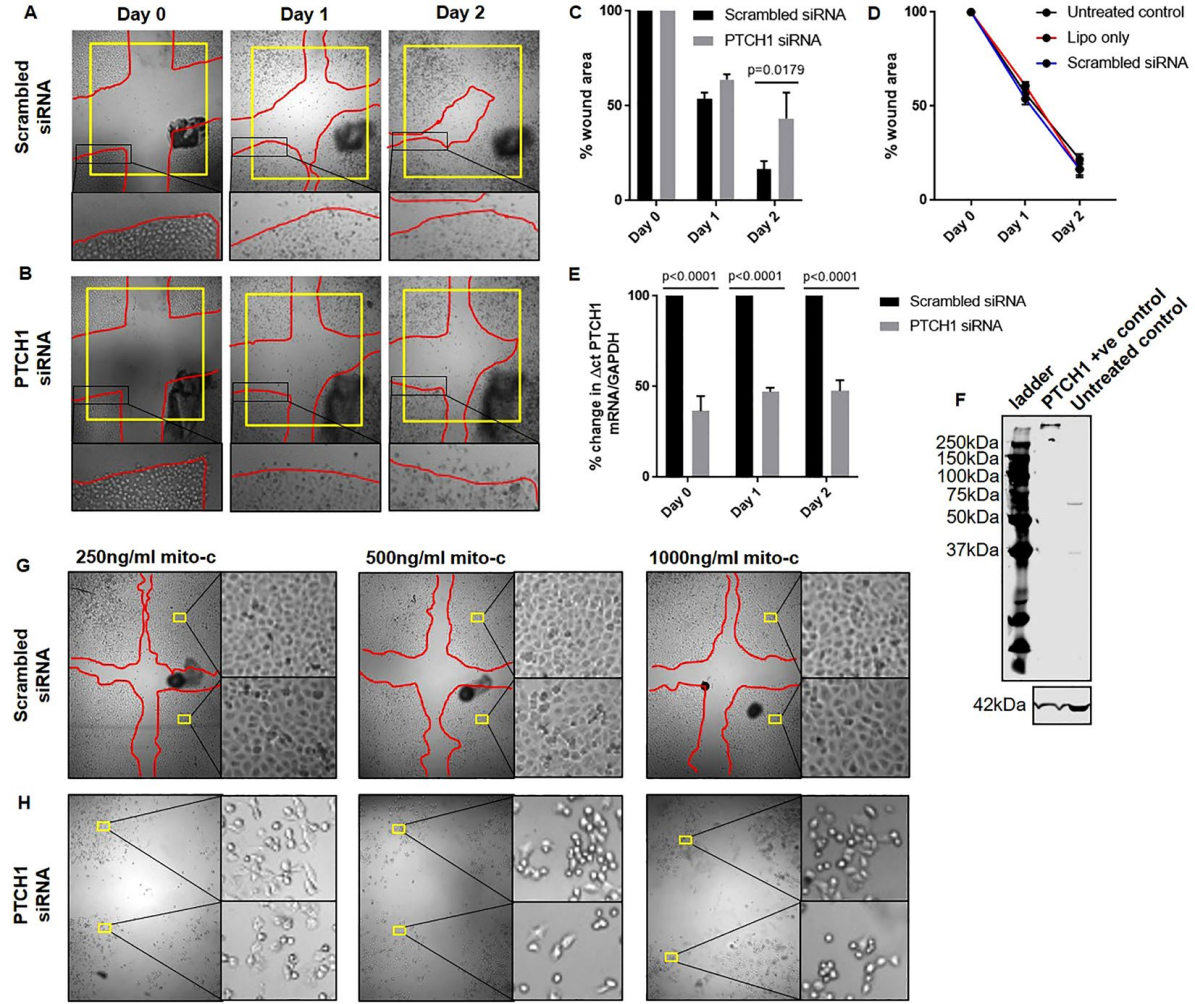
PTCH1 expression regulates cell proliferation and adhesion. Representative images of cross-hatched wounds in (**A**) scrambled siRNA- or (**B**) *PTCH1* siRNA-treated 1HAE_0_ cells imaged at day 0, 1 and 2 post-injury were shown. Red line indicated the wound edge boundary. Yellow box standardized the area of field. Black dots indicated pen marks of the same representative wound images taken over time. (**C**) Wound area remaining in scrambled siRNA- or *PTCH1* siRNA-treated cells was expressed as a percentage of the total area measured at day 0 in each individual wound. (**D**) Wound area remaining in untreated controls, lipofectamine (lipo)- and scrambled siRNA-treated cells was expressed as a percentage of the total area measured at day 0 in each individual wound. E) *PTCH1* mRNA expression in scrambled siRNA- and *PTCH1* siRNA-treated cells was normalized to 100% scrambled siRNA-treated cells for each time point. Values were expressed as mean ± SEM (N = 3–4 independent experiments). Two-way analysis of variance with Bonferroni’s multiple comparisons test was used in panel C,D. (**F**) Representative protein blot showing PTCH1 antibody expression and specificity in PTCH1-overexpressing lysate and untreated human airway epithelial cell lysate. Cell morphology was imaged 24 h post mitomycin C treatment in (**G**) scrambled siRNA- or (**H**) *PTCH1* siRNA-treated cells. Yellow boxes indicated 2 magnified regions in each condition. Red line indicated the wound edge boundary 24 h post injury showing normal wound closing ability in scrambled siRNA-treated cells.

To determine whether *PTCH1* is involved in cellular proliferation post-injury, monolayer “wound assays” were performed with the human airway epithelial cell line (1HAE_0_) after pre-treatment with scrambled or *PTCH1* siRNA (Fig. 4A,B). Silencing *PTCH1* expression delayed wound closure (% wound area filled on day 2 post-injury) compared to scrambled siRNA-treated cells (Fig. 4C). The dynamics of wound repair over time were not significantly different between non-treated controls, and cells treated with lipofectamine or scrambled siRNA (Fig. 4D). A single administration of *PTCH1*-targeting siRNA resulted in consistent knockdown in *PTCH1* mRNA expression compared to scramble-treated controls on day 0, 1 and 2 post-injury, respectively (Fig. 4E). A representative protein blot of PTCH1 antibody specificity on PTCH1-overexpressed lysate and untreated human airway epithelial cell lysate was shown (Fig. 4F).

To determine whether *PTCH1* is involved in cellular migration, 1HAE_0_ cells were pre-treated with mitomycin C (250, 500 and 1000 ng/ml) to inhibit cell proliferation by DNA cross-linking followed by treatment with scrambled or *PTCH1* siRNA. Silencing *PTCH1* expression dramatically decreased cell attachment 24 h post mitomycin C treatment compared to scrambled siRNA-treated cells (Fig. 4G,H). In summary, we conclude that *PTCH1* is required for normal human epithelial cell growth and attachment *in vitro*.

### *PTCH1* gene silencing attenuates EGF-induced *MUC5AC* expression *in vitro*

**Figure 5:**
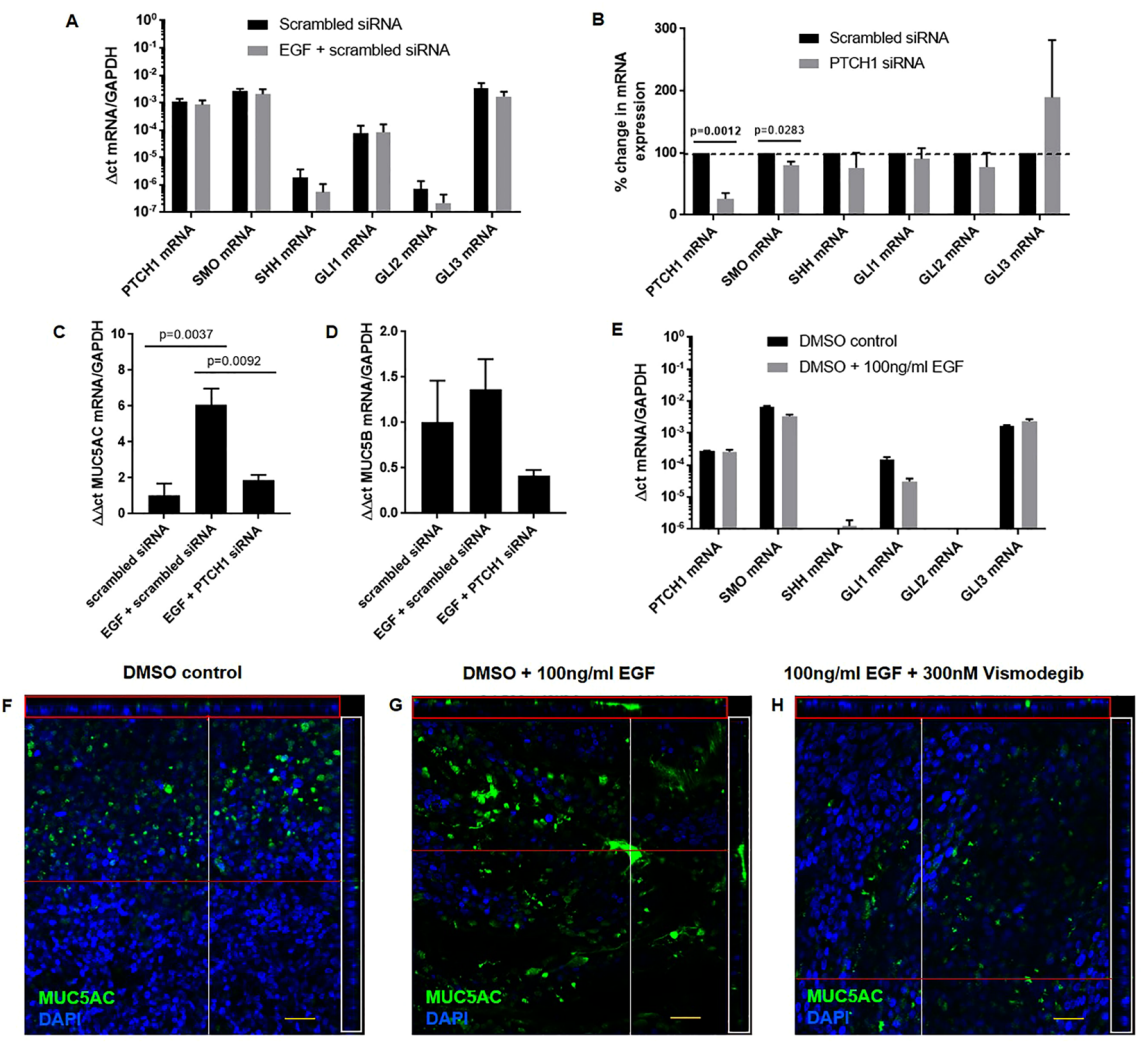
EGF-induced MUC5AC expression is mediated through PTCH1-SMO axis *in vitro*. (**A**) *PTCH1*, *SMO*, *SHH*, *GLI1*, *GLI2* and *GLI3* mRNA expressions were assessed in NCI-H292 cells treated with EGF only. (**B**) Percent change in *PTCH1*, *SMO*, *SHH*, *GLI1*, *GLI2* and *GLI3* mRNA expressions were assessed in NCI-H292 cells treated with scrambled or *PTCH1* siRNA for 48 h. (**C**) *MUC5AC* and D) *MUC5B* mRNA expressions were assessed in cells pre-treated with scrambled or *PTCH1* siRNA followed by EGF stimulation. Values were expressed as mean ± SEM (N = 3 independent experiments). A two-tailed unpaired parametric t-test was used for each gene in panels A,B. One-way analysis of variance with Bonferroni’s multiple comparisons test was used in panels C,D. (**E**) *PTCH1*, *SMO*, *SHH*, *GLI1*, *GLI2* and *GLI3* mRNA expressions were assessed in primary bronchial epithelial cells differentiated in ALI (N = 1 replicate). Immunofluorescence images showing goblet cell expression (MUC5AC) with nuclei counterstain (DAPI) in (**F**) DMSO vehicle, (**G**) DMSO + 100 ng/ml EGF, H) and 100 ng/ml EGF + 300 nM vismodegib (SMO inhibitor) (N = 1 replicate). Cross-sections through both axes of the membrane are shown by the red (x-axis) and white (y-axis) lines.

Human epidermal growth factor (EGF) has previously been used to induce MUC5AC expression in NCI-H292 cell line and in primary human bronchial epithelial cells differentiated in air liquid interface as a mucous induction model^16–18^. To determine whether *PTCH1* is linked to *MUC5AC* expression in epithelial cells, we used human epidermal growth factor (EGF) to stimulate *MUC5AC* mRNA expression in the NCI-H292 cell line treated with either a *PTCH1*-targeting or scrambled control siRNA. Notably, EGF stimulation did not alter the expression of hedgehog signalling pathway encoding transcripts *PTCH1, SMO*, *SHH*, *GLI1*, *GLI2* or *GLI3* (Fig. 5A). However, both *PTCH1* and *SMO*, but not *SHH, GLI1, GLI2* and *GLI3* mRNA expression were attenuated by ~75% and ~15%, respectively, in cells treated with *PTCH1* siRNA (Fig. 5B). The relative % change in mRNA expressions of *SHH*, *GLI1*, *GLI2* and *GLI3* remained unchanged after *PTCH1* gene silencing compared to scrambled siRNA-treated controls. Silencing *PTCH1* expression significantly attenuated EGF-induced upregulation of *MUC5AC* but not *MUC5B* transcripts (Fig. 5C,D).

Hedgehog signalling pathway-related gene expression levels in primary bronchial epithelial cells differentiated in ALI were comparable to those in NCI-H292 cell line at baseline (Fig. 5E). Similar to NCI-H292 cells, *SHH* mRNA expression was marginal detectable in primary ALI cultures. Representative confocal images revealed that treatment of ALI culture with 300 nM SMO inhibitor (Vismodegib) attenuated EGF-induced MUC5AC protein expression (Fig. 5F–H). Thus, we conclude that the expression of *PTCH1* is required and mediated through SMO activation for the expression of *MUC5AC* in response to EGF.

### *Ptch1*^+/−^ mice show reduced mucous expression in response to house dust mite

**Figure 6:**
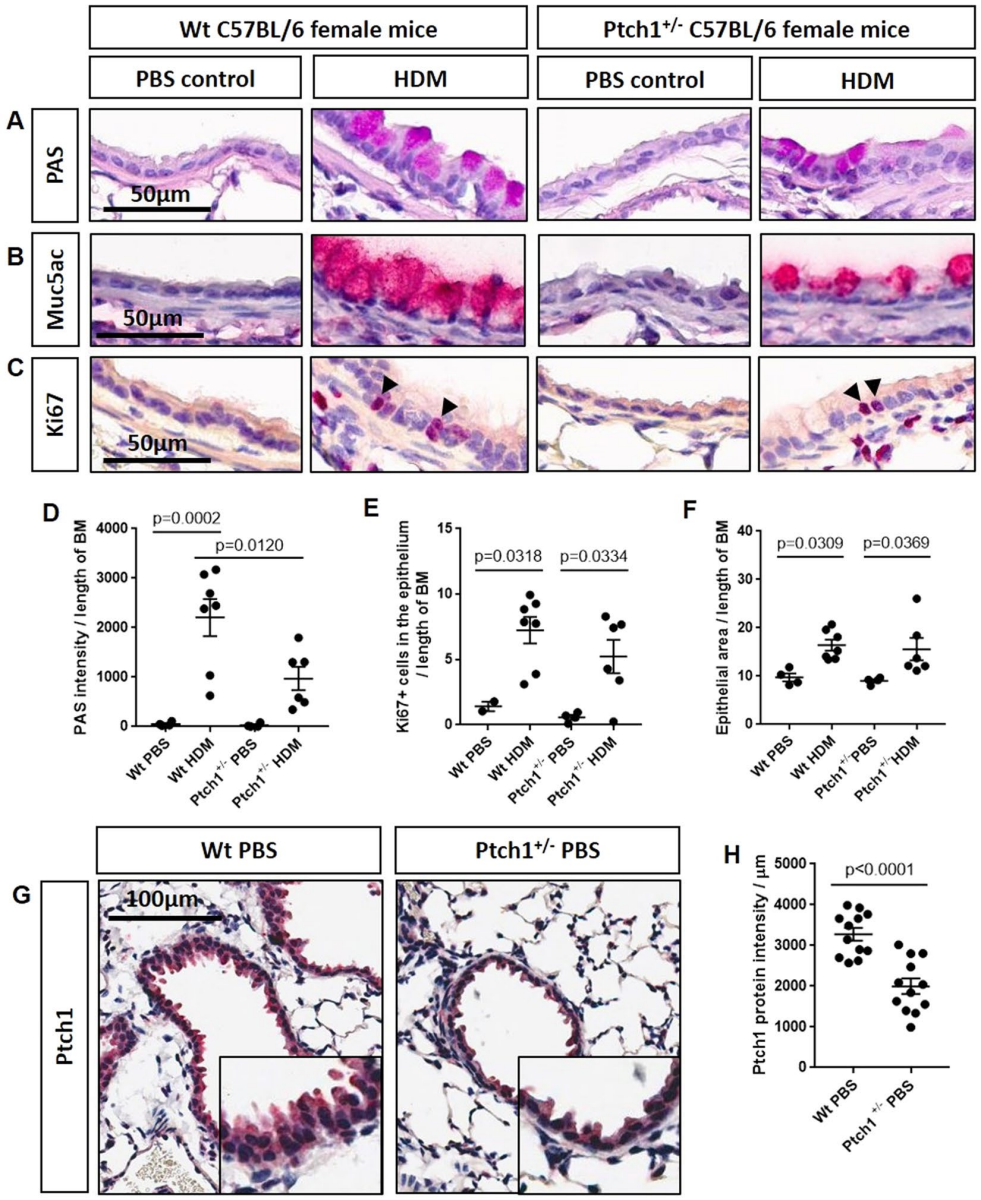
*Ptch1*^+/−^ mice were partially protected from HDM-induced mucous expression compared to HDM-exposed *Wt* mice. (**A**) Periodic acid Schiff (PAS)-, B) MUC5AC- and (**C**) Ki67-stained sections of paraffin-embedded mouse lung tissues from *Wt* and *Ptch1*^+/−^ mice after PBS or HDM exposures were shown. (**D**) Airway-specific PAS staining was normalized to length of basement membrane (BM). (**E**) Ki67+ epithelial cells and (**G**) airway epithelial thickness were normalized to length of BM. Scale bar = 50 µm. Values were expressed as mean ± SEM. One-way analysis of variance with Bonferroni’s multiple comparisons test was used in panels D–F. (**G**–**H**) Representative images and quantification of epithelial-specific Ptch1 protein expression in all visible distal airways in a representative Wt PBS- and *Ptch1*^+/−^ PBS-treated mouse.

We previously showed that chronic smoke exposure in a mouse model of COPD failed to induce the production of goblet cells in the airway epithelium^19^. Therefore, in order to evaluate the role of *Ptch1* in goblet cell expression, we used a house dust mite (HDM)-exposed mouse model^20–25^. Because homozygous *Ptch1*^−/−^ mice are embryonic-lethal^26,27^, we used *Ptch1*^+/−^ mice and wildtype (*Wt*) littermates and repeatedly exposed them to HDM (or a PBS vehicle control). Evaluation of mucous expression in lung airway epithelium by PAS histology revealed significantly reduced mucous production in HDM-exposed *Ptch1*^+/−^ mice compared to HDM-exposed *Wt* mice (Fig. 6A,D). Representative IHC images showed a reduction in MUC5AC protein expression in HDM-exposed *Ptch1*^+/−^ mice compared to HDM-exposed *Wt* mice, which were consistent with PAS expression (Fig. 6B). HDM exposure to *Wt* or *Ptch1*^+/−^ mice significantly increased Ki67+ cells in the epithelium (Fig. 6C,E) and epithelial thickness (Fig. 6F) to similar extent. IHC images showed PTCH1 protein expression was significantly reduced by ~50% in all measurable distal airway epithelium from representative Wt PBS and *Ptch1*^+/−^ mice (Fig. 6G,H). We conclude that *Ptch1* plays an important role in goblet cell expression and, when *Ptch1* expression is decreased, goblet cell expression is attenuated.

## Discussion

In the current study, we have identified the expression patterns and biological roles of PTCH1 in human lung tissue, epithelial cells and *Ptch1*^+/−^ mice. Our study demonstrates that PTCH1 protein is up-regulated in the airway epithelium of smokers (both current and ex-smokers) with COPD compared to non-COPD controls. We showed in our *in vitro* models that the attenuation of the *PTCH1* gene resulted in decreased cell proliferation and goblet hyperplasia, leading to reduced mucous production. This finding was also recapitulated *in vivo* with HDM-exposed *Ptch1*^+/−^ mice having reduced mucous expression compared to *Wt* mice. Taken together, these results suggest that up-regulation of PTCH1 in the COPD airway may contribute to mucous hypersecretion and the “chronic bronchitis” phenotype of COPD patients.

Although the link between mucous secretion and COPD progression is well documented, the precise mechanism by which PTCH1 is involved in COPD pathophysiology is not well understood. De Smet and colleagues showed that PAS+ cells on average were significantly elevated in the airway epithelium of smokers with moderate to severe COPD compared to never smokers and smokers without COPD^28^. MUC5AC is one of the most abundantly-expressed protein in the airway epithelium and has been shown to be regulated by several transcription factors including but not limited to NFκB, CREB, SPDEF, FOXA2, AP1 and SP1^29–33^. We used epidermal growth factor (EGF) to stimulate MUC5AC in our cell line because it has been shown that there is increased EGFR protein and EGFR-phosphorylation in lung epithelial cells of COPD patients^34,35^. Cigarette smoke-induced increase in *MUC5AC* expression was prevented by an EGFR-neutralizing antibody in NCI-H292 cell line^17^. Furthermore, EGF can also regulate the expression and secretion of sonic hedgehog via PI3K/Akt activation in isolated gastric parietal cells^36^. The direct biological contribution of PTCH1 to mucous expression has not been previously identified. Here, we showed that *PTCH1* gene silencing dramatically attenuated EGF-induced *MUC5AC* but not *MUC5B* mRNA expression in NCI-H292 cells. Recently, Kageyama-Yahara and colleagues showed that GLI, the principal family of transcription factors in hedgehog signaling, regulated MUC5AC transcription via direct protein-DNA interaction in the MUC5AC promoter region of human gastrointestinal cells^37^. These data reveal potential contributions of PTCH1 to EGF-induced increase in mucous expression in the airway epithelium of patients with COPD.

We have previously shown that chronic smoke exposure (6 months) of mice did not induce mucous expression in the distal airways compared to air-exposed controls, suggesting that it may not be an appropriate model to evaluate goblet cell metaplasia. To overcome this limitation, other groups have used ovalbumin or house dust mite (HDM) to reproducibly produce goblet cell metaplasia in mice. Although HDM exposure does not induce emphysema, approximately 15 to 30% of patients with COPD have overlapping features of asthma and thus this model may have relevance for the “bronchitic” features of COPD^38^. Likewise, using a mouse model of allergic airways disease, Le Cras and colleagues demonstrated that treatment with an EGFR tyrosine kinase inhibitor attenuated HDM-induced EGFR activation and goblet cell metaplasia in the airway epithelium^20^. Recently, Xu and colleagues showed that SHH neutralizing antibody or cyclopamine, (an inhibitor of SMO), attenuated eosinophil counts in BALF and mucous expression (PAS and MUC5AC staining) in ovalbumin-sensitized mice via up-regulation of *Spdef* and down-regulation of *Foxa2*, both of which are classical regulators of goblet cell metaplasia^39^. Consistent with their overall conceptual interpretation of their data and model, we showed that *Ptch1*^+/−^ mice exhibit reduced mucous-producing cells compared to *Wt* mice in response to HDM allergen challenge. Although we failed to detect SHH protein in bronchial alveolar lavage or serum of C57Bl/6-congenic *Wt* or *Ptch1*^+/−^ mice, previous studies have demonstrated an upregulation of SHH protein in whole lung homogenates of *Wt* BALB/c mice, a strain that typically responds more severely in HDM-induced allergic asthma^40^. In summary, these data provide novel evidence of crosstalk between EGFR-signaling and the PTCH1-SMO axis on airway mucous expression.

Although the precise biological implications of hedgehog signalling related to COPD remain to be fully clarified, several studies have demonstrated clinical relevance of PTCH1 and the hedgehog pathways to chronic airway diseases. In the context of asthma, Xu and colleagues showed that subjects with asthma exhibited elevated SHH protein expression in bronchoalveolar lavage fluid (BALF) compared to non-asthmatic control subjects^39^. Similarly, SHH protein was also shown to be increased in the BALF of patients with Idiopathic Pulmonary Fibrosis (IPF) compared to non-IPF subjects^41^. Consistent with this finding, Bolanos and colleagues showed that hedgehog signaling-related genes (*SHH*, *PTCH1*, *SMO*, *GLI1* and *GLI2*) were up-regulated in lung fibroblasts from patient with IPF compared to non-IPF control subjects^42^. In the context of COPD, both *HHIP* mRNA and protein were decreased in whole lung tissues from smokers with COPD compared to smokers without COPD^43^, suggesting an indirect activation of hedgehog signaling because HHIP has been shown to be a surface receptor antagonist to all three mammalian hedgehog homologs^44^. Furthermore, Figeac and colleagues showed that lung fibroblasts derived from COPD smokers expressed higher levels of activating peptide sonic hedgehog (SHH) and proteins related to the hedgehog signalling pathway compared to cells derived from COPD non-smokers^45^. Similarly, our data showed an indirect evidence of hedgehog activation accompanied by an increase in *PTCH1* mRNA and protein expression in the airway epithelium of patients with COPD, which has been shown to be induced via GLI family zinc-finger DNA-binding proteins^9,11,12^. In summary, these data suggest a strong contribution of hedgehog signaling activation in the lungs of patients with chronic airway diseases.

Small airways disease, which includes remodeling of the airway epithelium and sub-epithelial compartments is an important feature of COPD^46^. Hogg and colleagues showed that the airway epithelium and sub-epithelial components include lamina propria, smooth muscle and adventitia were significantly increased in patients with severe (GOLD 3) and very severe (GOLD 4 grades) COPD compared to healthy smokers without COPD^46,47^. Here, we showed that airway epithelial thickness, total epithelial cell count, Ki67+ epithelial cells were positively correlated with airway epithelial-specific PTCH1 protein expression in airway epithelial tissue in all subjects. Consistent with our finding, Liu and colleagues showed a progressive and significant increase in proliferation index (Ki67+ cell) in the airway epithelium of patients without and with COPD (moderate severity), respectively^48^. Our data further demonstrated that severe COPD patients have reduced Ki67+ cells in the airway epithelium compared to moderate COPD patients, which is suggestive of cellular exhaustion. Our *in vitro* data demonstrated that *PTCH1* gene knockdown significantly reduced wound closure, suggesting a role for cell proliferation. Our mouse model also showed a positive correlation between epithelial-specific Ptch1 protein expression with epithelial thickness and total epithelial cell count. In summary, these data indicate that hedgehog signaling may be associated with airway epithelial thickening and, in addition to other sub-epithelial compartments, contribute to the overall airflow limitation.

There are several important limitations to our study. This was a cross-sectional study, which prevented us from establishing any conclusion on the causality regarding the relationship between PTCH1 and mucous expression in the airways of patients with COPD. While we demonstrated in our *in vitro* and *in vivo* models that deficiency in *PTCH1* attenuated mucous expression, it is possible that other factors including but not limited to oxidative stress, local inflammatory response and microbial interactions may have activated the hedgehog signaling response in patients with COPD. Although our current epithelial-specific *PTCH1* mRNA and protein expression data showed paradoxical results from previous work where *PTCH1* mRNA expression was positively associated with FEV1/FVC in whole lung tissues from subjects with and without COPD^2^, it is likely that their whole lung tissues sampled from patients undergoing lung resection surgery were emphysematous regions containing a majority of alveolar tissues whereas we quantified airway epithelial-specific PTCH1 expression.

In summary, we showed that PTCH1 is increased in the airway epithelium of patients with COPD compared to those without COPD. Reduced expression of PTCH1 consistently attenuated mucous expression in our epithelial *in vitro* and *in vivo* models. For patients with COPD, mucous production and cough are two symptoms of the greatest burden and importance; yet current therapies have little or no effect on these endpoints^15^. This work provides new insights into COPD pathophysiology through connecting PTCH1 expression with mucous formation by the airway epithelium and nominates PTCH1 as a potential target of therapeutic intervention to reduce mucous producing-phenotypes in patients with COPD.

## Materials and Methods

### Human tissues and sample preparation

The lungs of five non-smoker controls, nine smokers with COPD GOLD STAGE 2 and eight smokers with COPD GOLD STAGE 3,4 biobanked within the James Hogg Lung Registry were used for histologic and immunohistochemical staining. The demographics of the subject lungs assessed are shown in Table 1. Age-matched human airway epithelial cells RNA samples from patients with or without COPD stored in our tissue biobank were used to measure *PTCH1* mRNA expression. The selected COPD patients were not taking inhaled corticosteroids at the time of the bronchoscopy. Patients without COPD consist of smokers and non-smokers that have undergone bronchoscopy for nodule or mass interrogation and/or with idiopathic chronic cough. Since these cells were previously expanded in monolayers in bronchial epithelial growth media (BEGM; Lonza) for only one cellular passage, gene expressions closely resembled those of the intact human airway epithelium. The demographics of the subjects where bronchial brushed were obtained are shown in Table 2.

Human lung tissue samples were obtained with informed consent from patients undergoing thoracic surgery as part of the James Hogg Lung Registry with approval of Providence Health Care Research Ethics Board (PHCREB) H00-50110. Human epithelial cells were obtained by bronchiole brushings from patients with informed consent who were undergoing bronchoscopy PHCREB protocol (H15-02166). All experiments in this manuscript were conducted in accordance with relevant guidelines and regulations approved by the University of British Columbia (B17-0027).

### Immunohistochemistry in human lung tissues

Formalin-fixed paraffin-embedded (FFPE) human lung tissues were stained with antibodies against PTCH1 (06-1102; EMD Millipore) and Ki67 (ab16667; Abcam) using the Bond Polymer Refine Red Detection kit on the Leica Bond Autostainer according to the manufacturer’s protocol. Slides were scanned using the Aperio imaging system (Leica Biosystem; Concord, Ontario). Airway epithelial-specific PTCH1 protein intensity was quantified using the Aperio imaging system, while the Ki67+ cells were manually counted and normalized to the length of the basement membrane.

### Immunofluorescence microscopy on human lung tissues

Formalin-fixed paraffin-embedded (FFPE) human lung tissue sections were dewaxed, antigen retrieved at pH6 and incubated with primary antibodies against PTCH1 (06-1102; EMD Millipore), MUC5AC (MA1-21907, ThermoFisher), and/or FOXJ1 (AMAB91254, Sigma) overnight at 4 °C and stained with Alex Fluor® 488 goat anti-mouse IgG and 594 goat anti-rabbit IgG (Life Technologies) for 2 h at room temperature. Slides were counterstained with DAPI (10236276001, Sigma), cover-slipped and visualized using confocal microscopy.

### Histologic assessment in lung tissues

Airway epithelial thickness and total airway epithelial cell count were measured in FFPE-human lung tissues stained with hematoxylin and eosin (H&E). Airway epithelial thickness was determined by normalizing the area enclosed between the apical surface and the basement membrane to the length of the basement membrane (BM). Total airway epithelial cell count was measured by counting the number of nuclei with normalization to the length of the BM. Total airway epithelial mucous expression was evaluated by staining FFPE-human lung tissues with periodic acid Schiff (PAS). PAS-positive cells were quantified with normalization to the length of the BM. Luminal contents (i.e. mucous plugs, luminal exudates and portions of sloughed off epithelium) were excluded from the airway epithelial-specific measurements. All airways <2 mm in cross-sectional diameters were evaluated per subject. All measurements were quantified using the Aperio ScanScope software.

### Cell culture

Human airway epithelial cell line (1HAE_0_) was obtained from Dr. Dieter Gruenert University of California, San Francisco^49^ and cultured in DMEM (Gibco BRL; Invitrogen, Carlsbad, CA) with 10% fetal bovine serum (FBS). To determine whether PTCH1 is involved in wound closure, 1HAE_0_ cells were pre-treated with scrambled or *PTCH1* siRNA (ThermoFisher Scientific; Waltham, MA, USA) for 24 h. After a complete medium refresh, cells were allowed to grow to 100% confluence for another 24 h prior to wounding. Cross-hatched wounds were created per well using a sterile p200 pipet tip followed by imaging at day 0, 1 and 2 post-wounding to monitor wound closure. Images were taken at 10X magnification and wound edges were manually traced at each time point and normalized to the matched original wound area at day 0 using Image J software (NIH, USA).

To demonstrate whether PTCH1 gene silencing affects DNA synthesis and cell proliferation, 1HAE_0_ cells were pre-treated with silencer-select scrambled siRNA (4390843; ThermoFisher Scientific) or *PTCH1* siRNA (4392420; ThermoFisher Scientific) for 24 h. After a complete medium refresh, cells were allowed to grow to 100% confluence for another 24 h. Cells were pre-treated with 250, 500 and 1000 ng/ml of mitomycin C (M4287, Sigma) for 2 h prior to wounding as described above.

Mucous-producing human mucoepidermoid pulmonary epithelial carcinoma (NCI-H292; ATCC) were cultured in in RPMI (Gibco BRL; Invitrogen, Carlsbad, CA) with 10% FBS. Cells were cultured in reduced serum (1% FBS) for 24 h prior to experiments. Cells were pre-treated with silencer-select scrambled siRNA (4390843; ThermoFisher Scientific) or *PTCH1* siRNA (4392420; ThermoFisher Scientific) for 24 h. The media was replaced, and cells were stimulated with 10 ng/ml of human epidermal growth factor-EGF (585508; BioLegend) for another 24 h.

Methods of cell culture in air liquid interface were previously described with modification^50^. In brief, human bronchial-brushed epithelial cells from a healthy non-smoker was cultured in T25 flasks in Bronchial Epithelial Growth Media (BEGM, Lonza) and sub-cultured when 90% confluent. Cells at 21 days post ALI were pre-treated with DMSO (vehicle control) or 300 nM vismodegib (GDC-0449, Selleckchem) for 5 days followed by treatment of 100 ng/ml EGF for 7 days with fresh complete media replaced 3 times per week.

### Real-time PCR

Total RNA samples were extracted using the RNeasy mini plus kit (74136; Qiagen) and reverse-transcribed into cDNA using the iScript cDNA synthesis kit according to the manufacturer’s protocol (1708891; Biorad). Real time PCR was performed on a CFX384 Touch Real-Time PCR Detection System (Biorad) using human TaqMan gene expression assays (ThermoFisher Scientific) to measure *PTCH1* (Hs00181117_m1), *SMO* (Hs01090242_m1), *SHH* (Hs00179843_m1), *GLI1* (Hs00171790_m1), *GLI2* (Hs00257977_m1), *GLI3* (Hs00609233_m1), *MUC5AC* (Hs00873651_mH) and *MUC5B* (Hs00861595_m1) mRNA expressions with normalization to *GAPDH*. Gene expression was expressed as ΔΔct fold changes with normalization to control values, and Δct when multiple genes are displayed in parallel to show relative abundance.

### Western blot

Methods on western blot analysis was previously described with modification^50^. In brief, membranes were incubated with primary antibodies against PTCH1 (06-1102; EMD Millipore) and beta-actin (A1978, Sigma) followed by incubation of IRDye® 800CW Goat anti-Mouse IgG (A-11005, ThermoFisher) and IRDye® 680RD Goat anti-Rabbit IgG (A-11001, ThermoFisher) for 2 h at room temperature, and visualized using LI-COR Odyssey CLx (LICOR, Lincoln, Nebraska USA). HEK293T cell lysate overexpressing human PTCH1 (LY424834, Origene) as positive control and human airway epithelial cells were used to confirm PTCH1 antibody specificity.

### Immunofluorescence microscopy on differentiated cell culture

Apical ALI culture compartment was gently washed with PBS to remove excess mucous secretion and fixed with 10% phosphate-buffered formalin for 30 min at room temperature. Cells were permeabilized with 0.5% Triton X-100 followed by blocking with serum-free protein block (X090930–2, Agilent/DAKO) for 1 h at room temperature. Membranes were washed with PBS 3 times for 5 min each followed by incubation with primary antibody against MUC5AC (MA1–21907, ThermoFisher) overnight at 4 °C. On the following day, membranes were washed with PBS 3 times for 5 min each followed by incubation of IRDye® 800CW Goat anti-Mouse IgG (A-11005, ThermoFisher) for 2 h at room temperature. Membranes were washed 3 times as described above, detached with a fine scalpel and placed the membrane with cells facing up on a microscopic glass slide. Nuclei were counterstained with DAPI, coverslipped and imaged using confocal microscopy.

### Mice

*Ptch1*^*tm1Mps*^/J (JAX 00308) were purchased from The Jackson Laboratory (Bar Harbor, ME) and were maintained by heterozygous backcross to C57Bl/6 J mice at the Biomedical Research Centre specific-pathogen free transgenic facility. Female mice of 10–16 weeks were used for experiments. Experimental procedures were approved by the Animal Care Committee (Protocol# A15-0113) of the University of British Columbia based on guidelines provided by the Canadian Committee on Animal Care.

#### HDM treatment protocol

HDM extracts (endotoxin < 2500 EU/mg) (Greer Lab, Lenoir, NC) were prepared in phosphate-buffered saline (PBS) and then 40 µl volumes were administered to deliver intranasally 100 µg HDM extract/mouse for three consecutive days (days 0, 1 & 2) and then 20 µg/mouse on days 19, 20, 21 & 22^51^. Twenty-four hours after the last exposure (day 23), lungs were lavaged, and blood and lung tissues were collected.

### Immunohistochemistry in mouse lung tissues

Formalin-fixed paraffin-embedded (FFPE) mouse lung tissues were stained with antibodies against Ptch1 (06-1102; EMD Millipore) and Ki67 (ab16667; Abcam) using the Bond Polymer Refine Red Detection kit on the Leica Bond Autostainer according to the manufacturer’s protocol. Formalin-fixed paraffin-embedded (FFPE) mouse lung tissues were stained with antibody against MUC5AC (MA1-21907, ThermoFisher) using the high sensitivity universal detection system-MACH kit (Biocare Medical, Concord, CA) as previously described^50^.

Total airway epithelial mucous expression was evaluated by staining FFPE-mouse lung tissues with PAS staining. Total epithelial-specific PAS intensity and epithelial thickness were normalized to the length of the BM. Airway epithelial-specific Ki67+ positive cells and Ptch1 protein intensity were normalized to the length of the BM. All slides were scanned using the Aperio imaging system and color intensities were quantified using the Aperio ScanScope software.

### Statistical Methods

Data were tested for normality prior to the selection of a parametric (normal distribution) or Mann Whitney (non-normal distribution) t-test, Kruskal Wallis multiple comparisons test, one-way ANOVA with Bonferroni’s multiple comparisons test, and linear regression test, where appropriate. All data were analyzed using GraphPad Prism version 6 (GraphPad Software Inc, San Diego, CA, USA) and were expressed as mean ± SEM. Statistical significance was considered at P < 0.05.

## Data Availability

The datasets generated during and/or analysed during the current study are available from the corresponding author on reasonable request.
